# Retraction Note: Zinc Deficiency Causes Glomerulosclerosis and Renal Interstitial Fibrosis Through Oxidative Stress and Increased Lactate Metabolism in Rats

**DOI:** 10.1007/s12011-026-05294-0

**Published:** 2026-08-13

**Authors:** Zixuan Huang, Yajie Liao, Yunxi Zheng, Shang Ye, Qianyu Zhang, Xiaohong Yu, Xiaoxin Liu, Ningxu Li

**Affiliations:** 1 Department of Nephrology, Liyuan Hospital, Tongji Medical College, Huazhong University of Science and Technology, Wuhan, Hubei China; 2 Department of Urology, The First Affiliated Hospital, Jiangxi Medical College, Nanchang University, Nanchang, Jiangxi China; 3 Queen Mary University of London, London, E1 4NS UK

**Retraction Note: Biological Trace Element Research (2024) 203:2084–2098**

**https://doi.org/10.1007/s12011-024-04306-1**

The Editors-in-Chief have retracted this article. Concerns were raised regarding a number of figures, specifically:

- Figure 2c: the lower section of MASSON/NC, 100X and the upper section of MASSON/ZS, 100X appear to overlap;
- Figures 3e and 4c: the LDHA band of Figure 3e and the p-AKT band of Figure 4c appear to overlap, when flipped;
- Figure 4f: sections of AKT/NC and N-cadherin/NC appear to overlap;
- Figures 4f and 5c: the N-cadherin/NC panel of Figure 4f and the PAI-1/ZS panel of Figure 5c appear to overlap.

The authors have stated that original data are unavailable. The Editors-in-Chief therefore no longer have confidence in the reliability of the data reported in the article.

Authors Zixuan Huang, Shang Ye, and Qianyu Zhang agree with this retraction and Authors Yajie Liao, Yunxi Zheng, Xiaohong Yu, Xiaoxin Liu, and Ningxu Li did not respond to correspondence from the Publisher about this retraction.

